# The GENIUS Grid Portal and robot certificates: a new tool for e-Science

**DOI:** 10.1186/1471-2105-10-S6-S21

**Published:** 2009-06-16

**Authors:** Roberto Barbera, Giacinto Donvito, Alberto Falzone, Giuseppe La Rocca, Luciano Milanesi, Giorgio Pietro Maggi, Saverio Vicario

**Affiliations:** 1 Istituto Nazionale di Fisica Nucleare, Sezione di Catania, Via S. Sofia 64, I-95123 Catania, Italy; 2 Department of Physics and Astronomy of the University of Catania, Viale Andrea Doria 6, I-95125 Catania, Italy; 3 Dipartimento Interateneo di Fisica di Bari Via E. Orabona 4, I-70126 Bari, Italy; 4 NICE S.r.l. – Via Marchesi di Roero 1, I-14020 Cortanze (AT), Italy; 5 CNR – Institute for Biomedical Technologies – Via Fratelli Cervi 93, I-20090 Segrate (MI), Italy; 6 INFN Sezione di Bari Via E. Orabona 4, I-70126 Bari, Italy; 7 CNR – ITB Bari – Via Amendola 122D, I-70126 Bari, Italy

## Abstract

**Background:**

Grid technology is the computing model which allows users to share a wide *pletora *of distributed computational resources regardless of their geographical location. Up to now, the high security policy requested in order to access distributed computing resources has been a rather big limiting factor when trying to broaden the usage of Grids into a wide community of users. Grid security is indeed based on the Public Key Infrastructure (PKI) of X.509 certificates and the procedure to get and manage those certificates is unfortunately not straightforward. A first step to make Grids more appealing for new users has recently been achieved with the adoption of robot certificates.

**Methods:**

Robot certificates have recently been introduced to perform automated tasks on Grids on behalf of users. They are extremely useful for instance to automate grid service monitoring, data processing production, distributed data collection systems. Basically these certificates can be used to identify a person responsible for an unattended service or process acting as client and/or server. Robot certificates can be installed on a smart card and used behind a portal by everyone interested in running the related applications in a Grid environment using a user-friendly graphic interface. In this work, the GENIUS Grid Portal, powered by EnginFrame, has been extended in order to support the new authentication based on the adoption of these robot certificates.

**Results:**

The work carried out and reported in this manuscript is particularly relevant for all users who are not familiar with personal digital certificates and the technical aspects of the Grid Security Infrastructure (GSI). The valuable benefits introduced by robot certificates in e-Science can so be extended to users belonging to several scientific domains, providing an asset in raising Grid awareness to a wide number of potential users.

**Conclusion:**

The adoption of Grid portals extended with robot certificates, can really contribute to creating transparent access to computational resources of Grid Infrastructures, enhancing the spread of this new paradigm in researchers' working life to address new global scientific challenges. The evaluated solution can of course be extended to other portals, applications and scientific communities.

## Background

**Figure 1:**
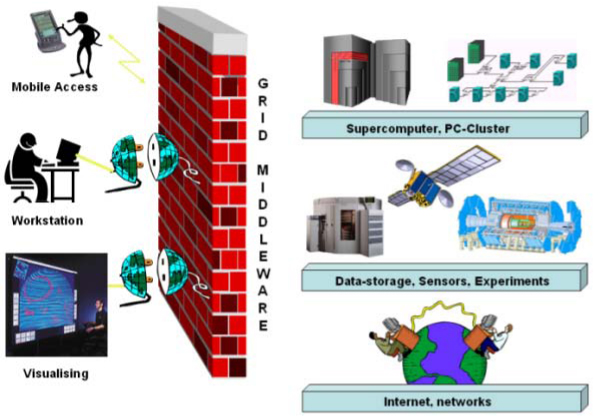
**Grid vision for e-Science**. Transparent, ubiquitous and collaborative access to the Grid.

In the last few years the needs of many researchers have become more and more demanding. Recent progresses in several scientific domains and the new challenges scientists have to face, have made it essential to devise platforms able to ensure appropriate support to complex multi-discipline research activities. Modern scientists need to access distributed computational and storage resources and start collaborative work in order to address common problems. Grid technology, based on open standards protocols, is the technology which permits an efficient sharing and management of a wide range of heterogeneous computational resources such us: supercomputers, storage systems, data resources and instruments, regardless of their geographical location. *"A computational grid is a hardware and software infrastructure that provides dependable, consistent, pervasive and inexpensive access to high-end computational capabilities" *[1]. The massive potential of Grid technology is becoming indispensable for many scientific and industrial applications belonging to different domains such as: Astronomy, Computational Chemistry, Earth Science, Financial Simulation, High Energy Physics and Biomedicine. In the life science domain for example, today, technology has brought biological information to grow at an impressive rate. The huge computational resources provided by Grid technology are particularly necessary, for example, in searching the human genome or to carry out simulations of molecular dynamics for the study of new drugs. Researchers in the field of the Earth science need to manipulate a lot of data in order to create complex models to predict weather forecasts, river floods and earthquakes. The paradigm introduced by this new technology is conceptually not unlike electrical grids. In an electrical grid, wall outlets allow users to link to an infrastructure of resources that generate, distribute, and bill for electricity. When a user connects to the electrical grid, he doesn't need to know from where the power comes from. Grid computing uses *middleware *(a virtual layer which allows the user to run his application in a specific grid resource in a transparent way) to coordinate disparate IT resources across a network, allowing them to function as a virtual whole. In order to allow scientists to perform new challenging researchers expediting the production of scientific results, e-Infrastructure tools and services need to become features of researchers' everyday working life. In other words, they should just *see *the grid as a seamless extension of their own workstation for what concerns both job execution/monitoring and data access/management. What a generic non-expert user would like to do is just to access the grid services in a transparent way from everywhere using one of the different electronic devices such us: desktop, laptop, PDA, last generation of mobile phone, etc.), as he does with the World Wide Web (figure 1).

Unfortunately the scenario we have today is a bit different. Due to the ongoing evolution of this technology so far no standard is available and there's an initial gap scientists need to overcome before to start up. Moreover each Grid user needs to subscribe for a personal X.509 certificate, adhere to a specific VO and obtain an account on one of the trusted UI (User Interface) for the project where he is involved. All these steps may have caused fear and confusion amongst researchers and caused the running away of potential new users. The Italian INFN [2] and the Italian web technology company NICE [3] Srl, at the beginning of 2002, started to develop the GENIUS [4,5] Grid Portal in order to provide transparent access to the Grid for the end-users. Thanks to this work, today researchers coming from different scientific domains can access the Grid to run their own applications using a conventional web interface. All the complexity of the underlying *gLite *grid *middleware *[6] will be hidden to the end-user by the portal. In this manuscript we are going to introduce the new feature designed in this portal in order to support robot certificates.

## Methods

### Robot certificate

Starting from the 28^th ^of Feb. 2008 the Italian INFN CA (Certification Authority) [7] modified its CP/CPS *(Certification Policy and Certification Practice Statement of a Certification Authority) *to permit users to apply for robot certificates which now are officially recognized as a standard by the IGTF (International Grid Trust Federation) [8]. UK and Netherlands CAs are already issuing robot certificates. The exploitation of these certificates by other CAs in the next few years is warmly foreseen. These new certificates have been introduced to permit users, who are not familiar with deal personal certificates and belonging to a VO, to experience the Grid paradigm for research activity reducing the initial barriers. The robot certificate (also known as *portal *certificate), associated with a specific application that the user wants to share with the Grid community, can be installed in a smart card and used with a portal by anyone who is interested in running this application in a Grid environment using an user-friendly interface. For security reasons, in order to reduce the risks of having the portal certificate compromised, the INFN CA decided to issue these new certificates on the *Aladdin eToken *smart card [9]. Each smart card can support several robot certificates: one for each application we want to share with other users of the same VO. An user's PIN is prompted every time the certificate stored on the smart card is read to generate a *proxy*. The proxy is a term used to describe a certificate that is derived from, and signed by, a normal X.509 Public Key Certificate. It is used to grant access within a PKI based authentication system. Use of a proxy credential is a common technique used in security systems to allow entity A to grant to another entity B the right for B to be authorized with others as if it were A. In other words, entity B is acting as a proxy on behalf of entity A.

**Figure 2:**
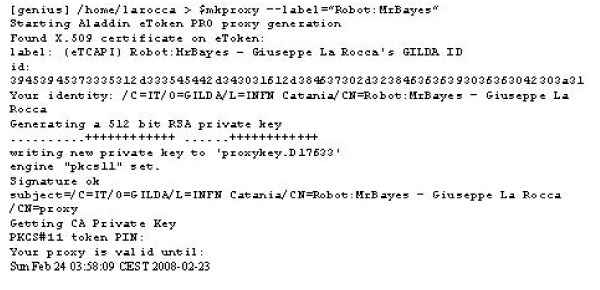
**Creation of a plain proxy**. Generation of a user proxy using the mkproxy script.

Once the robot certificate is safely stored on the eToken smart card, the mkproxy shell script developed at NIKHEF [10] can be used to generate grid proxies reading the certificate directly from it. This script requires some special programs and libraries, which need to be installed before attempting to use it. Starting from a single robot certificate installed on a Aladdin eToken PRO, a user can generate a plain grid proxy by issuing the command as shown in figure 2.

**Figure 3:**
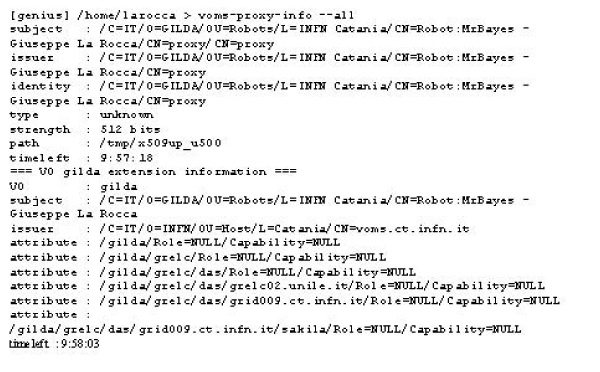
**Adding the needed extension to the proxy before to use it**. Add the VOMS extension to the plain proxy.

In this example a plain proxy is signed by the robot certificate stored on the eToken with the given label Robot:MrBayes. Once the plain grid proxy is generated, it is possible to add the VOMS (Virtual Organization Management Service) [11] extensions by issuing the command voms-proxy-init using the noregen option as shown in figure 3.

With the mkproxy script a proxy certificate is generated for the user. This proxy is used to access Grid and run applications. In this work we have extended the architecture of the portal by adding the functionality introduced by this script. As the proxy certificate has been created the user can start to access the Grid. Since the beginning, the adoption of a personal certificate to access Grid resources has represented a limiting factor for the real spreading of this paradigm. Many researchers would be interested in using Grid as a tool to resolve problems and speed up the creation of scientific results, but the basis of the PKI risks to discourage many of them. The benefits introduced by robot certificates in Life Science are far reaching because they can contribute to make transparent the access in Grid of biologists interested to run some specific applications.

In the next sub-sections the architecture of the GENIUS Grid Portal, powered by EnginFrame, will be presented and the work carried out to extend its framework to support these new certificates will be described in detail.

### The GENIUS Architecture

**Figure 4:**
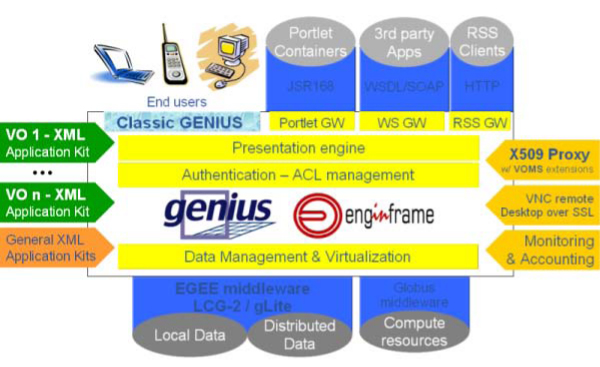
**EnginFrame architecture (copyright^© ^NICE Srl)**. The Web-based technology developed by the Italian company Nice Srl that enables the access and the exploitation of Grid-enabled applications and infrastructure hiding all its complexity to the end-users.

GENIUS portal, based on EnginFrame framework, has an architecture as shown in figure 4:

• The client side (top left in the figure) represented by a user's workstation running a web browser. Thanks to recent modern client side technologies, many kinds of devices can be used in addition to usual notebooks or workstations, like palmtops or new generation of mobile phones;

• the protocols (top right in the figure): the users can use different protocols to access the presentation engine over services, the exposed gateways are available for portlets, web services and RSS; at the present, these protocols can be used by third party clients and not vice versa, accessing the virtualized services;

• the server side (right in the figure): a UI machine (equipped with the LCG/gLite middleware services able to submit jobs and manage data on the Grid) which runs the Apache Web Server, the Java/XML portal framework EnginFrame [12], developed by NICE Srl, and GENIUS itself. The server block is composed by:

◦ the presentation engine for the rendering of layouts and XSL/XML streams, based on leading WEB standards, provides access to underlying services via https, including html, soap and RSS; also the XML virtualization layer provides a set of XML processing functions that simplify the management of information coming from plug-in extensions;

◦ the layer for the Authentication and ACL (Access Control List) management, a core component, with many options to restrict the views of services to different profiles of users, influencing the behaviour of other services;

◦ the Data Management and Virtualization layer provides an abstraction of access to remote data and sources and support for a complete data life-cycle;

• The Application kits (left centre side): make the abstraction layer that hides the business logic of specific end-user applications, on the right hand side (right centre side) other transversal services that allow the VOMS Proxy authentication by user X509 certificate, the access to X11 interactive application using VNC [13] over SSL in secure way, and Monitoring; the Applications are developed by plug-in extensions, and GENIUS code itself is developed like a plug-in to the EnginFrame core;

• The remote resources (bottom right in the figure): the Grid, computational resources and distributed data;

Briefly, thanks to the Agent-Server design of the EnginFrame core, the EF Server manages the end user browsing by providing web pages via https, talks to the EF Agent, expects XML response from the Agent; on the other side, the EF Agent translates requests from the EF Server into actions on the computing resources, (i.e. on the gLite User Interface), with the right credentials and correct *user-id *on the machine, and translates the response from the UI into XML. Using the EnginFrame services the user can interact with files on the UI and, from there, the user can send jobs to the Grid and manage the data of the given Virtual Organization the user belongs to. The use of the web interface eliminates any problem connected to the need of a particular Operating System and/or middle-ware running on the client, and to the locations themselves of the client and the server: the user can interact with the grid from everywhere and with "everything". Making use EnginFrame capability of services virtualization, GENIUS is transparently compliant with latest versions of the LCG [14]/gLite middle-ware, and can be easily installed on a variety of Linux flavours, ranging from RedHat 9 to Scientific Linux, both 32 and 64 bits platforms. The multi-layered architecture of EnginFrame greatly simplifies the development of Web Portals exposing computing services that can run on a broad range of different computational Grid systems. In the last few years the architecture of the portal has been successfully customized to run applications of different scientific domains such as: Life Science, Humanities, Earth Science, Astro-Particle Physics, HEP. Due to its modularity architecture of the EnginFrame framework it is considered a Grid *gateway*.

### Accessing the Grid using a robot certificate and the GENIUS Grid Portal

The Service Definition Files (SDF™) are the core of the EnginFrame framework. Basically they are simple high-level XML files which describe how to link the existing command-line world to users' Web interface. Each SDF must have an *.xml *extension and, in order to be processed, must be included in the DOC_ROOT of the Web Server. Behind the Web Server, data is managed through the Spooler abstraction. A Spooler is a dedicated zone in the file system. It's used for hosting files provided by users (e.g., input files) or generated by other services (e.g., output or temporary files).

**Figure 5:**
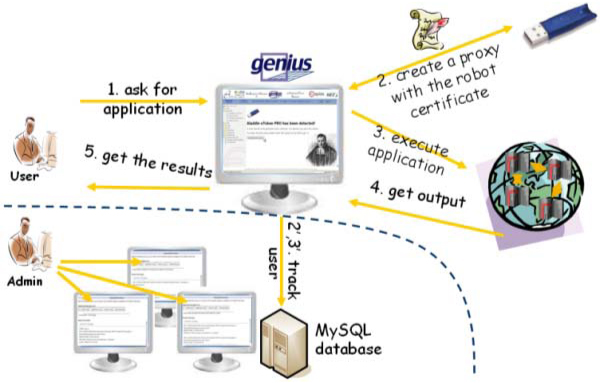
**The GENIUS Grid Portal and robot certificates**. This is the new scenario which is now available on board of the GENIUS Grid Portal to access Grid Infrastructures using robot certificates.

In this work one of these SDF has been modified to enable the features introduced by these new certificates with the portal and generate proxy certificates from the robot one. If no robot certificates are available on the server, the normal authentication based on a Java applet which request a personal user's certificate will be performed. The generation of the proxy certificate starting from a robot one is performed in a transparent way for the end-user. Thanks to this work the new scenario depicted in the figure 5 is now possible.

If the smart card is available on the server, an automatic service, deployed in the portal, will drive the user to create a temporary proxy before running the application connected with the robot certificate in Grid. Hereafter follows the action invoked from the portal to generate the proxy using the robot certificate.

**Figure 6:**
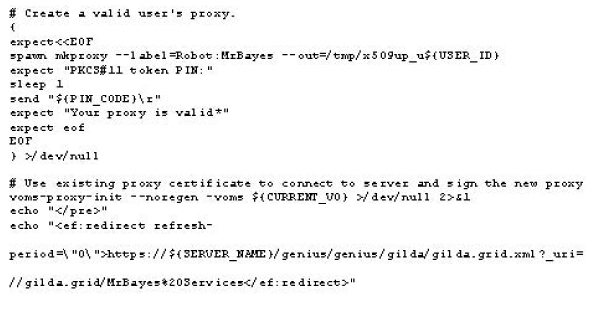
**Creation of the user proxy from the GENIUS Grid portal**. The figure shows the action performed from the portal every time a user logs to the portal. The generation of the user proxy is completely transparent for the end-user.

After proxy creation, the user is automatically redirected to the home page of the application connected with the certificate. Any other attempt to access unauthorized services will be blocked by the portal. Any request submitted by the user via the portal will be performed in the Grid in a transparent way according to the workflow described in figure 6. All the complexity of the underlying middleware is completely hidden by the portal. When the results about the computations are available, the user can retrieve them using a conventional web browser.

### Users Tracking System (UTS)

**Figure 7:**
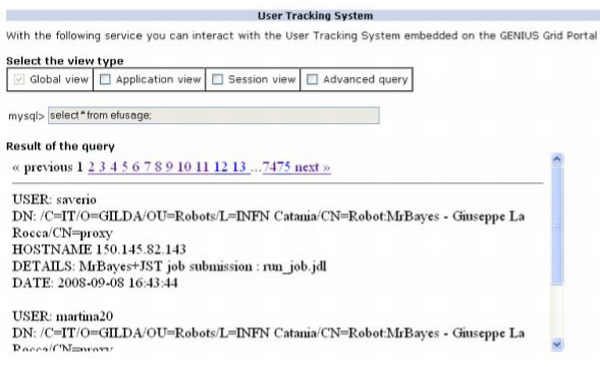
**The Users Tracking System (UTS)**. The accounting system developed on GENIUS Grid Portal to collect and monitor the user's payload produced in Grid.

Since with this approach the access to a Grid infrastructure is opened to a wide basin of users (just a simple account on the server where the portal runs is needed), in order to enhance the security and monitor the payload produced by users who access the Grid using the portal certificate, an UTS has been designed and deployed in the GENIUS architecture. Based on an underlying MySQL database and on PHP, JavaScript and XML code, the system registers the user's payload generated in Grids by the robot certificate. So far the system has been instructed to catch the following events: opening/closing of user' session and job submission instances. All the accounting data collected by UTS can be examined only by the administrator. Several ad-hoc services, with a restricted policy, have been designed in the portal so as to allow the administrator to interact with the UTS using a web interface only. With the introduction of this additional features it is possible to monitor, in each moment, who is working and what they doing on the grid resources. Figure 7 shows an example of accounting data collected by the system.

Four different system views to inspect the accounting data produced in Grid by a robot certificate are available:

• a *"global view" *which allows the administrator to retrieve a complete dump of all the information registered in the database;

• a *"session view" *which reports only information about all the sessions started and closed by the users;

• an *"application view" *which reports information about the application submission;

• an "*advanced query*" which allows administrator to perform some advanced queries by putting a specific "*where" *clause in the dedicated text area.

## Results

The GENIUS Grid portal that transparently supports robot certificates has been successfully used by non-grid users, involved in the context of the LIBI Italian Laboratory for Bioinformatics [15] to run a bioinformatics application on a Grid Infrastructure. In this section some details about the application and its workflow which has been set up in order to run this application on the EGEE [16] Grid Infrastructure are shown. The application MrBayes (Ronquist and Huelsenbeck 2003) [17] produces a Bayesian phylogenetic inference among different aligned biosequences. The inference allows identifying the distribution of the most likely genetic relationship among the set of chosen biosequences and at the same time the best set of values for the parameters of the postulated model of evolution of the biosequences. MrBayes has a great richness of model of evolution for DNA (both as nucleotide and codon), RNA (model for evolution of doublet of nucleotide to model the secondary structure of an RNA molecule), protein, and even arbitrary hereditary discrete characters. Another peculiarity of the application is that it allows the usage of mixed models, such as using different models for different parts of each biosequence with the possibility to share parameters among the different models.

The program uses a Metropolis-Coupled Monte Carlo Markov Chain (MCMCMC) to perform the Markovian integration necessary to solve numerically the Bayesian equation. The MCMCMC approach allowed the development of a parallel version of the algorithm (Altekar *et al. *2004) [18]. The result of the numerical integration is a sample from the posterior distribution allowing interesting development for future grid implementation. In fact different samples of the posterior distribution could be merged together to increase reliability of the results and to check for the convergence of the algorithm. But it should be noted that the program is not perfectly scalable given that for moderately complex problems the time necessary to reach stationary, and to produce useful sampling, is not so small compared to the maximum time allowed in each single CPU of EGEE.

The input required is a single text file, nexus formatted (Maddison *et al. *1997) [19], subdivided in a data block and MrBayes block in which the models and the parameter of Markovian integration are defined. The output is composed of three kinds of large files (typically of several hundreds of mega base each) that describe, respectively, the posterior distribution of numerical and topological parameters, and several diagnostic measures related to the mixing of Markov chains and the converging of the algorithm as whole.

**Figure 8:**
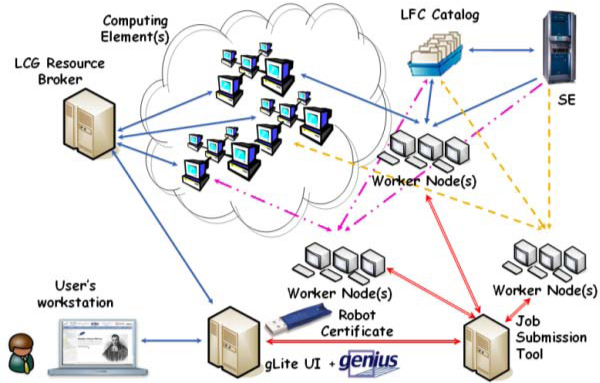
**MRBAYES: A simple bioinformatics use's case**. The figure depicts the application workflow which has been set up to permit bioinformatics to run Bayesian Phylogenetic Inference on a large scale.

**Figure 9:**
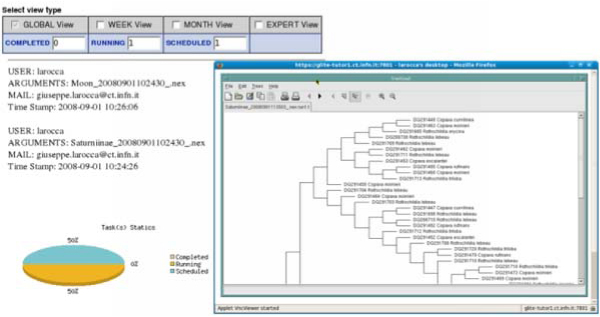
**Monitoring and Visualization services**. The figure shows how user can monitor his tasks interacting with the JST and display phylogenetic trees using TreeViewX[27]. Visit also the official TreeViewX web site:

The client side of the application submission workflow (figure 8) is represented by a user's workstation running a web browser. The server side is represented by a gLite UI machine, equipped with the latest gLite middleware services to submit jobs and manage data on Grid, the Apache Web Server, the Java/XML portal framework EnginFrame developed by NICE Srl and the GENIUS Grid Portal itself. After the user has logged in, a proxy certificate is requested by the portal in order to access the distributed resources of a Grid Infrastructure according to the GSI [20] standard. If the Aladdin eToken PRO 32 Kb with he robot certificate is available in the server, it will be used by the portal to generate the needed proxy for the user before granting access to the Infrastructure. This operation is completely transparent for the end-user. In a few minutes the robot certificate stored on the usb token is read to generate the proxy certificate. Once the proxy certificate has been successfully created, the user is automatically redirected to the home page of the application. Thanks to the services developed with the portal the user can provide input settings for the application before submitting its parallel version in Grid. Moreover, in order to improve the reliability of the application workflow and deal with possible job failures a JST (Job Submission Tool) [21], developed by INFN Bari, has also been introduced into the architecture. This tool has been adopted for the submission of large number of jobs in an almost unattended way. It is based on the concept of "task" to be executed. The entire problem is first subdivided into elementary tasks: then all the tasks are inserted into a DB server. In the submission phase all the jobs are identical, in fact when the job is submitted it does not know which task has to be executed. Only when the job lands and starts executing on a WN does it request the central DB a task to execute. Information on the execution of each task is logged in the central DB. Only if all steps are correctly executed by the job, is the status of that particular task on the central DB updated to "Done". In this way the central DB provides a monitoring of the task execution and no manual intervention is required to manage the resubmission of the failed tasks: tasks which are found in a "running" state after a given time interval, are considered failed and automatically reassigned to new jobs. The tool is also able to provide the monitoring of any kind of debugging information that could be useful to understand how the jobs are going. The Database schema used to monitor jobs on the grid allows the definition of the information to be reported at run-time in a completely dynamic way, as it is based on the concept of "Variable" and "Value". The tool has been used with success for the submission of the order of hundreds of thousands of jobs to the Grid required by specific applications and can also be used for exploiting all the computational resources available on the Grid. Figure 9 shows the monitoring and the visualization system developed for the bioinformatics application.

## Conclusion

The present work aims at reporting the work performed by the Italian INFN in order to adopt robot certificates in e-Science. This work demonstrates how it's possible to access and exploit the massive potential of grid technology without worrying about the complexity of the GSI authentication. The benefits introduced by this work are far-reaching for several user communities and applications. The valuable results depicted in this work can be easily extended to other scientific domains and different applications. The GENIUS Grid Portal and its features is the official portal of the GILDA t-Infrastructure [22] for Grid dissemination and training set up. It is managed by INFN in the context of the EGEE Projects, but some other regional Grid projects such as Trigrid [23] and PI2S2 [24] are adopting the GENIUS portal with success, porting on the web interface many applications running on their infrastructure, being such a powerful gateway to the grid resources with the required security. The solution evaluated and described in this manuscript is not of course restricted to the GENIUS Grid Portal and can be easily extended to other portals.

## Competing interests

The authors declare that they have no competing interests.

## Authors' contributions

All the authors prepared the manuscript. GL and AF modified GENIUS architecture to support robot certificate and the UTS. GL implemented web-based services on the GENIUS Grid Portal to permit users to run Bayesian Phylogenetic Inference on a large scale. GD implemented the Job Submission Tool and contributed to designing the UTS. GM contributed to the design and the implementation of the UTS prototype on a previous GENIUS release. SV collaborated to the development of the wrapper for MrBayes within the Job Submission Tool and defined the test cases. All authors contributed to the discussion and have approved the final manuscript.
